# Suppressing Interfacial Instability of Immiscible Liquid‐in‐Liquid Flow Using Magnetic Forces

**DOI:** 10.1002/advs.202510327

**Published:** 2025-11-07

**Authors:** Arvind Arun Dev, Gholamhossein Bagheri, Eberhard Bodenschatz, Thomas M. Hermans, Bernard Doudin

**Affiliations:** ^1^ IPCMS UMR 7504 Université de Strasbourg, CNRS 23 Rue du Loess Strasbourg 67034 France; ^2^ Laboratoire Colloïdes et Materiaux Divises, CNRS UMR 8231, Chemistry Biology & Innovation, ESPCI Paris PSL Research University 10 rue Vauquelin Paris 75 005 France; ^3^ Laboratory for Fluid Physics Pattern Formation and Biocomplexity, Max Planck Institute for Dynamics and Self‐Organization 37 077 Göttingen Germany; ^4^ Institute for Dynamics of Complex Systems University of Göttingen 37 077 Göttingen Germany; ^5^ Laboratory of Atomic and Solid State Physics Cornell University Ithaca NY 14 853 USA; ^6^ Sibley School of Mechanical and Aerospace Engineering Cornell University Ithaca NY 14 853 USA; ^7^ IMDEA Nanociencia C/ Faraday 9 Madrid 28 049 Spain

**Keywords:** droplets, ferrofluid, instability, interfaces, liquid–liquid

## Abstract

Interfacial instability prevents a liquid jet from flowing indefinitely within air or another liquid. An approach is presented here to suppress interfacial instability by means of a magnetic force applied by a ferrofluid envelope around the jet. The stability limits occurring within a large parameter window are experimentally investigated with length and time scales governed by the magnetic Bond number. Instabilities can be generated by modifying the magnetic force strength externally, with a remarkable return to stability when removing the external stimulus. The current system with soft and slippery interfaces enables investigations of flow systems beyond the limits of standard hydrodynamics allowing for exciting applications in flow chemistry, interface engineering and transport of biological materials.

## Graphical abstract

This paper investigates the magnetic stabilization of an immiscible inner liquid surrounded by a ferrofluid sheath. Suppressing instabilities and thermomagnetic manipulation of the inner liquid are shown. The results provide insight into Ferrohydrodynamic jet control for applications of industrial, pharmaceutical and physiological relevance.

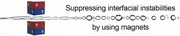

## 1 Introduction

Hydrodynamic instability imparts irreversibility to the state of flow. A classical ubiquitous problem is a liquid cylinder that breaks up into droplets to minimize the surface energy, i.e., the Rayleigh–Plateau Instability (RPI).^[^ ^1^ ^]^ Tuning the RPI is of crucial importance in inkjet printing,^[^ ^2^, ^3^ ^]^ micro fabrications,^[^ ^4^ ^]^ coating stability, pesticide spraying, fog harvesting, soft materials,^[^ ^5^ ^]^ and emulsion production. Overcoming the RPI is required for drug delivery with reduced viscous drag,^[^ ^6^ ^]^ optimization of hydropower,^[^ ^7^ ^]^ delicate biomaterial manipulation and 3D printing.^[^ ^8^ ^]^ To overcome interfacial instabilities in general, tuning the relative contribution of competing gravitational, surface, and viscous dissipation energies is required.^[^ ^9^, ^10^, ^11^ ^]^ External surface contributions like curvature,^[^ ^12^, ^13^ ^]^ hydrodynamic slip,^[^ ^14^ ^]^ non‐Newtonian liquids,^[^ ^15^ ^]^ nanostructure coating,^[^ ^16^ ^]^ elastic,^[^ ^17^ ^]^ electrostatic^[^ ^18^ ^]^ and ferro‐hydrodynamic interactions^[^ ^19^ ^]^ can further help in tailoring the growth of interfacial instabilities. However, completely avoiding the growth of instability remains a fundamental challenge.

We have recently shown that magnetic encapsulation—by balancing magnetic and surface tension forces—^[^ ^20^ ^]^ results in stability of a “static” liquid cylinder (of potentially any shape^[^ ^21^ ^]^), which otherwise is unconditionally unstable. However, the underlying physics governing the limits of instability is complex and unresolved. The typical length and time scale of such stabilization and their application in droplet microfluidics are of fundamental as well as engineering importance, but unknown. We show here how a magnetically controlled interface between two immiscible liquids can be tuned, here we have an immiscible glycerol and oil‐based ferrofluid system,^[^ ^22^ ^]^ resulting in stable coflowing liquids.^[^ ^21^ ^]^

## 2 Results

### 2.1 Stability of Liquid‐in‐Liquid Confinement Under Flow Conditions

**Figure 1:**
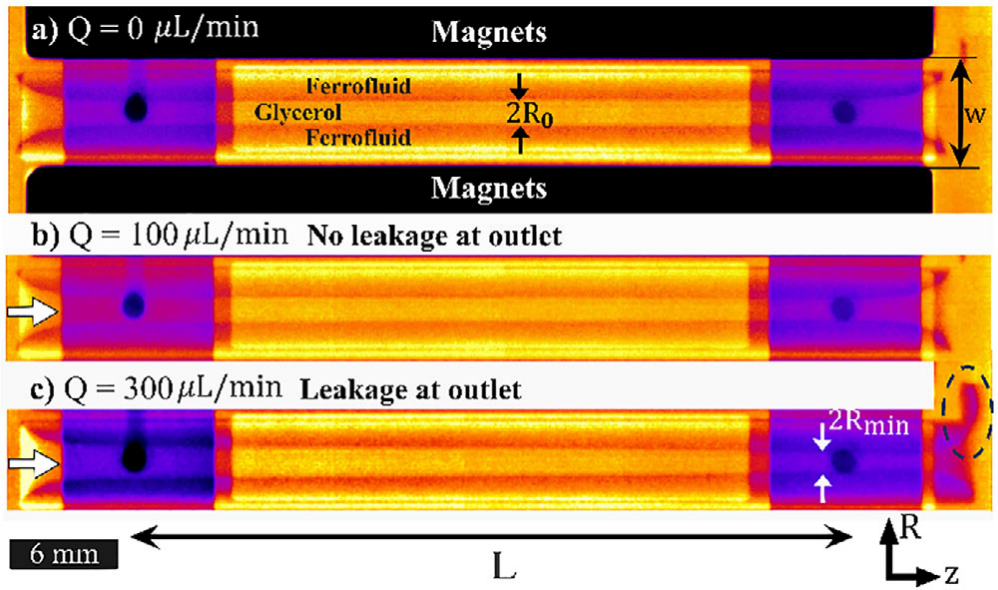
X‐ray radiography of magnetically encapsulated cylindrical flow channels. Glycerol is the inner flowing liquid and APG1141 is the surrounding ferrofluid. a) No flow case *Q* = 0 µL/min with 2*R*_0_ as the no flow diameter, b) stable flow at *Q* = 100 µL/min, c) unstable (ferrofluid egress at outlet) state for *Q* = 300 µL/min. *L* is the length of the cylindrical section and 2*R*_*min*_ is the diameter of the glycerol flow at *z* = *L*. The curved section at the inlet and outlet is due to the fringe magnetic field.

We use an axisymmetric flow channel design,^[^ ^20^, ^23^ ^]^ where the ferrofluid concentrically encapsulates the non‐magnetic liquid cylinder (glycerol) of diameter 2*R*_0_ and length *L* (**Figure** **1** **a**), under a quadrupolar external magnetic field designed as approximately linear in radial direction and invariant in azimuthal direction.^20^ The X‐ray radiography image is shown in Figure 1 with bright (glycerol) and dark (ferrofluid) bands. The flow is set using a syringe pump, and a liquid‐in‐liquid flow is realized, and the system is then imaged using a homemade X‐ray setup with resolution ± 20 µm.

**Figure 2:**
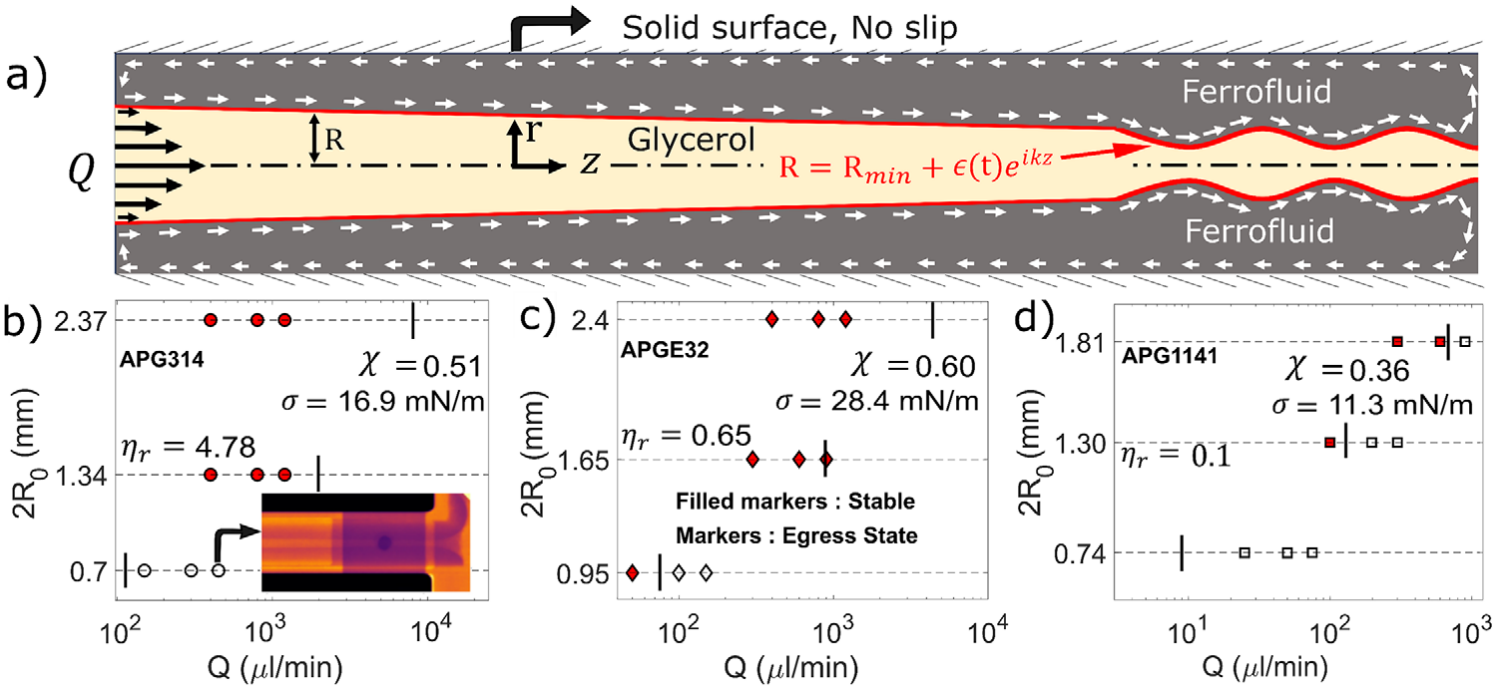
Stability range of axisymmetric magnetically confined flow channel. By varying the diameter, the relative viscosity of the two liquids (*η*_*r*_ = *η*_*g*_/*η*_*f*_), interfacial tension (σ) and the flow rate (*Q*). a) Schematic for linear stability analysis under the small slope assumption with perturbation at the outlet of the kind *R* = *R_min_* + *ε(* *t*)*e^ikz^*. Experiments with filled markers and open markers denote experimentally stable and unstable egress ferrofluid states, respectively. b) Glycerol‐APG314 with *η*_*r*_ *= 4.78*, the inset shows a case where ferrofluid egress takes place. c) Glycerol‐APGE32, *η*_*r*_ *= 0.65*, d) Glycerol‐APG1141, *η*_*r*_ *= 0.10*. The vertical line corresponds to *Q_limit_* calculated analytically from RPI instability criteria, using Equation 3.

In contrast to conventional liquid‐in‐liquid architecture via flow focusing, it is only the inner liquid (non‐magnetic) that has a defined flow rate (Figure 1b), and the momentum is transferred to the encapsulating sheath (ferrofluid) layer through interfacial shear. Beyond a critical flow rate (*Q_limit_*), ferrofluid leaks are the first indication of the destabilization of the liquid–liquid interface (compare Figure 1b,c dashed circle). For increasing flow rate, these liquid cylinders (glycerol) encapsulated by ferrofluid act as a soft material,^[^ ^21^ ^]^ and deform in response to flow (see Figure 1c) from inlet to outlet (also schematically in **Figure** **2** **a**), complicating the stability calculations. It is required to pinpoint the physics governing *Q_limit_* for various combinations of magnetic pressure, shear force, and the destabilizing Laplace pressure. In engineering terms, we need a state diagram showing *Q_limit_* as a function of flow *Q* and no‐flow diameter 2*R*_*0*_ for different ferro‐hydrodynamic properties. Figure 2 shows the state diagram data differentiating stable and egress/unstable states (dark ferrofluid moving adjacent to magnets, seen in Figure 2b) inset for ferrofluid APG314 and in Figure 1c for ferrofluid APG1141) for three ferrofluids of different magnetic susceptibility *χ* and viscosities *η*_*f*_, encapsulating a liquid (glycerol) of viscosity *η*_*g*_ and fluid interfacial tension *σ* between ferrofluid and glycerol. Experimental data (markers: egress/unstable state and filled markers: stable state) in Figure 2b–d indicate that *Q_limit_* decreases with 2*R*_0_ and *η*_*r*_ = *η*_*g*_/*η*_*f*_ . This is because the Laplace pressure becomes more important at smaller diameters, at which viscous shear increases and magnetic pressure decreases.^[^ ^21^ ^]^

In the Stokes regime (*Re*<<1), below *Q_limit_*, the inner liquid cylinder has unique properties, with no equivalent we are aware of in an artificially engineered system: there is no lower bound to *Q_limit_* (dripping failure) because even the static system is stable; the stabilization persists up to an infinite axial length, in contrast to flow focusing; the flow remains stable against external perturbations of up to 250 mL min^−1^ of water where *Re*>>1. Hence, the liquid–liquid interface is stable in both viscous and inertia dominated regimes (see video V1, Supplementary Video for oscillating flow input, and Figure S1, Supporting Information for further details). Beyond *Q_limit_*, the interface destabilizes at *R* = *R_min_*. Note 2*R_min_*, is the diameter at the output (*z* = *L* in Figure 1c and *cf*. Figure 2a), linked to the flow rate *Q* and the no‐flow diameter 2*R*_*0*_. The latter is set by experiments, filling the cavity with the flowing inner liquid (here glycerol), and then slowly adding the desired amount of ferrofluid to concentrically envelope the inner liquid. 2*R_min_* is the “weakest point” along the flow direction at the end of the flow channel, where the shear force is maximum and the magnetic pressure is minimum. In the design of Figure 1, it is near the outlet of the system, with deformation of the interface under flow making it smaller than 2*R*_0_.^[^ ^21^ ^]^ The balance of the shear force, the magnetic force and interfacial tension results in the deformation profile $\frac{\partial D}{\partial z}=\frac{-32\eta_{g}Q}{\pi D^{4}\beta \left[\frac{M_{r}}{\pi w}A\left(1+B\right)-\frac{\sigma }{D^{2}}\right]}$ with *D*  =  2*R* and volume conservation boundary condition^[^ ^21^ ^]^ $\int_{0}^{L}R^{2}\;dz=R_{0}^{2}L.$ Here *M_r_* = 1.26 T is the remanent magnetization of magnets and *w* is the distance between the magnet pair, respectively (See Figure 1a and ref. [21] for magnetic design). *A* and *B* are known functions of the magnetic field at the inner liquid‐ferrofluid interface governing the relation *M*(*H*), where *M* and *H* are magnetization of the ferrofluid and the applied magnetic field, respectively. They are derived by fitting a polynomial to the magnetization curve *M*(*H*)^21^. *β* is the drag reduction parameter (calculated) for the lubricated system (see Figure S2, Supporting Information). The 2*R_min_* value is typically ≈ *R*_*0*_ for stable states in our experiments.

Figure 2b–d summarizes the state diagram for the stability of our liquid‐in‐liquid flow using different ferrofluids with engineering parameters 2*R*_*0*_ and *Q*. The vertical line denotes the calculated *Q_limit_* for conditional stability (see details below). Note that most experiments show stability persisting very near the calculated flow limit, indicating that ferrofluid shearing effects are not exceedingly limiting our experiments stability range. We emphasize that stability is ensured up to high flow rates (as compared to typical values in microfluidics), in addition to indefinite stability at the smallest flow rates—that is, completely eliminating the dripping failure.

A quantitative stability model was achieved for viscous flows (*Re*<<1) under the linear stability framework (Figure 2a), with perturbation at the weakest point of the type *R* = *R*_*min*_ + *ε*(*t*)*e* ^*i* *kz*^  where *R_min_* is the minimum radius of the cylindrical flow channel (glycerol) at the outlet (at *z* = *L*) and $\frac{\varepsilon }{R_{min}}\ll 1$. Note from Figure 2, there is also a linear term (*‐cz*) in the perturbed state, i.e *R* = *R_min_* + *ε*(*t*)*e^ikz^* − *cz*, where *c* is the slope of deformation. In the linear stability analysis, the slope is small and hence, the problem can be treated locally by neglecting the linear term (see Section S3,Supporting Information for scaling of slope). The stability of the liquid–liquid interface in this deformed state (Figure 2a) is guaranteed if the growth rate of the disturbance ${\frac{\partial \varepsilon (t)}{\partial t}|}_{R_{\min}}$ is negative for all perturbation wave numbers *k*. For a linear magnetic medium (*M = χH*), *d* *ε*/*d* *t* obeys Equation 1 (See Figure S2, Supporting Information)

(1) $${\left.\frac{\partial \varepsilon \left(t\right)}{\partial t}\right|}_{R_{min}}=\frac{\varepsilon \left(t\right)R_{min}\beta_{min}\sigma }{16\eta_{g}}k^{2}\left[1-R_{min}^{2}k^{2}-\frac{16\mu_{0}\chi \left(\chi +1\right)M_{r}^{2}R_{min}^{3}}{\pi^{2}w^{2}\sigma }\right]$$

Here *M* is the magnetization of ferrofluid in an applied magnetic field of magnitude $H=\frac{4M_{r}R}{\pi w}$. $k=\frac{2\pi }{\lambda }$ is the wave number where *λ* is the wavelength of perturbation. *β*_*min*_ is the drag reduction parameter calculated at *R_min_*. It depends on the viscosity ratio *η*_*r*_ and the function of the radius of flow, ferrofluid thickness. The disturbance has a solution of a kind *ε*(*t*)∝*e* ^*t* *τ*(*k*)^ with

(2) $$\tau \left(k\right)=\frac{-R_{min}\beta_{min}\sigma }{16\eta_{g}}k^{2}\left[R_{min}^{2}k^{2}-1+\frac{16\mu_{0}\chi \left(\chi +1\right)M_{r}^{2}R_{min}^{3}}{\pi^{2}w^{2}\sigma }\right]\;$$

Here *τ*(*k*) is the growth rate of perturbations that now depends on the magnetic parameters as well. Note that in the absence of a magnetic force (*χ* = 0), the last term in *τ*(*k*) will disappear and *τ*(*k*) > 0 for small *k*, namely, an exponential growth of *ε*(*t*) makes the interface unstable, as expected. In this case, the fastest growing disturbance is of wavelength $\lambda_{max}=2\pi R_{min}\sqrt{2}\approx 9R_{min}$, as reported in the RPI literature^[^ ^14^, ^24^ ^]^ (see Section S3, Supporting Information for details). However, when considering the magnetic force, there is conditional stability of the liquid–liquid interface, *τ*(*k*) < 0 for all *k* if:

(3) $$2R_{min}>\frac{1}{2^{1/3}}{\left(\frac{\sigma \pi^{2}w^{2}}{\mu_{0}\chi \left(\chi +1\right)M_{r}^{2}}\right)}^{1/3}\;\;\;$$

Note that Equation 3 also resembles the condition derived for the stability of a soft‐solid cylinder of diameter 2*R* against Plateau–Rayleigh instability,^[^ ^9^, ^10^ ^]^ where stability requires $2R>\frac{\sigma_{s}}{E}$, with *σ*_*s*_  and *E* are the solid surface tension and elastic modulus, respectively. Comparing with Equation 3 gives the elasticity estimates of the liquid–liquid interface. It is essentially a ratio of interfacial tension and the resistive magnetic force. The resistive magnetic force acts as a conservative part like an elastic behavior for any *R* ≥ *R_min_*. *At* small *R*, with linear magnetic media (*M* = *χ* *H*), the elasticity can be mapped as *E*_*m* − *lin*_ *=* $\frac{2\mu_{0}\chi \left(\chi +1\right)M_{r}^{2}R^{2}}{\pi^{2}w^{2}}$. At large *R*, where the ferrofluid is magnetically saturated (*M* = *M_S_*), the apparent elasticity expression will depend on *M_S_*.^[^ ^21^ ^]^ This re‐affirms that the magnetically confined liquid–liquid interface can be considered as ultra‐soft.^[^ ^21^ ^]^

**Figure 3:**
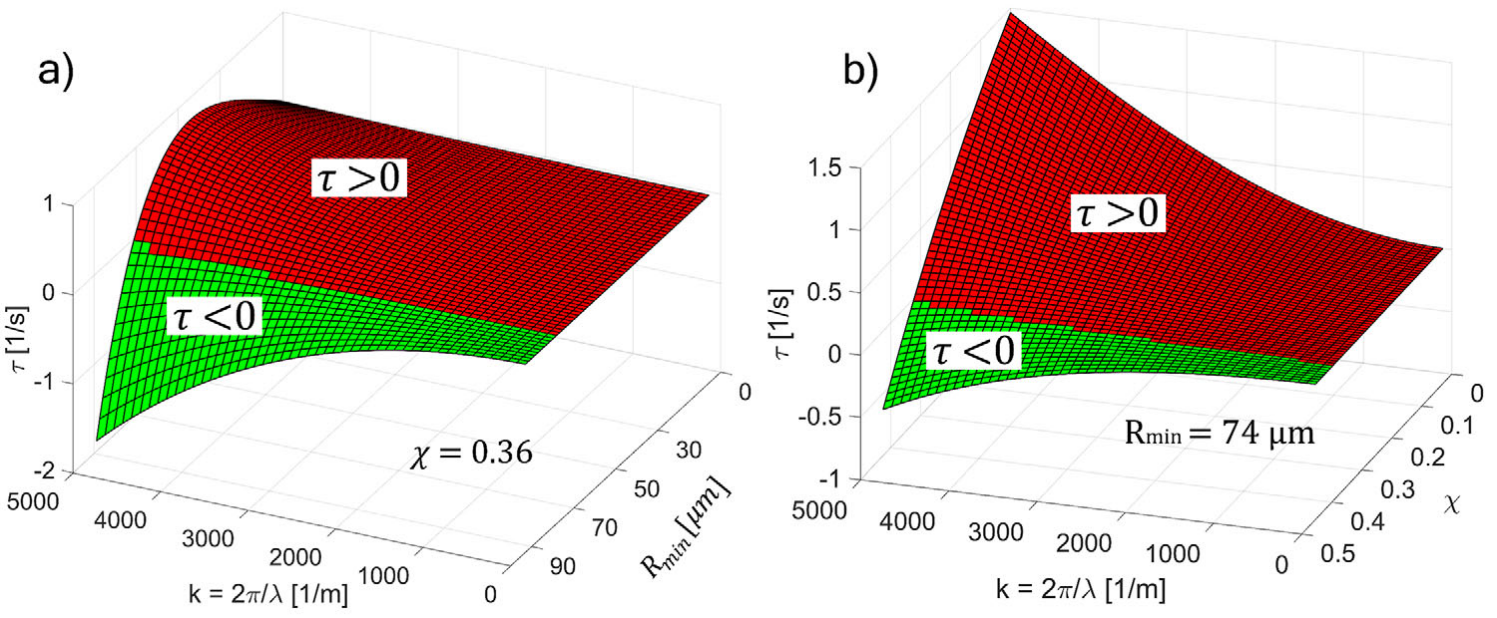
Dispersion relation *τ*(*k*). a) For a specific case of glycerol flow encapsulated with APG1141 ferrofluid of magnetic susceptibility *χ* = 0.36. b) Dispersion relation for variable *χ* and a fixed size, *R_min_* = 74 µm. Red and green zones depict the increasing and decreasing growth rate, respectively. Where *k* and *λ* are the wave number and wavelength of the disturbance.

For the experimental condition in Figure 2d—that is, the APG1141‐glycerol system (*σ* = 11.3±  0.15 mN m^−1^, *χ* = 0.36)—the marginally stable state is *R_min_* ≈ 74 µm following Equation 3. **Figure** **3a** shows the dispersion relation *τ*(*k*)  for APG1141 and illustrates that *τ* <  0 (green) persists when *R* > *R_min_* for all *k* values, which is the requirement for claiming flow stability. At the output of the encapsulated flow, the extremities of the external magnets cause a reduced (stray) magnetic field. We model this as a reduction of the ferrofluid magnetic susceptibility *χ* and illustrate in Figure 3b how this triggers instability. For *R_min_* ≈ 74 µm, the interface can be destabilized if *χ* is reduced below 0.36 (i.e., the susceptibility of APG1141 ferrofluid), for at least one *k*, which will be used to switch between stable/unstable (see below).

Introducing $\lambda_{max}=2\pi R_{min}\sqrt{2}$ and *Γ* = 64*η*_*g*_ *R_min_*/*σ* *β*_*min*_ as the fastest growing wavelength and timescale (*Γ* = 1/*τ*_*max*_) for the system without magnetic force, we can introduce the typical length and time scales as $\lambda_{m-max}=\lambda_{max}/\sqrt{1-Bo_{m}}$ and *Γ*_*m*_ = *Γ*/(1 − *Bo_m_*)^2^ for the system with magnetic force field^[^ ^25^, ^26^, ^27^ ^]^ (see Section S2, Supporting Information for details). Here, $Bo_{m}=\frac{16\mu_{0}\chi \left(\chi +1\right)M_{r}^{2}R_{min}^{3}}{\pi^{2}w^{2}\sigma }\;$ is the magnetic Bond number that determines the relative contribution of magnetic and surface energies. Equation 3 can be simply put as *Bo_m_* > 1 for stability, with the fastest growing wavelength imaginary. However when *Bo_m_* < 1, the fastest growing wavelength is finite and real, with length and timescale that depend on *Bo_m_*. This explicitly shows how to tune the dynamics with the magnetic confinement (*w*), magnet strength (*M_r_*) and ferrofluid magnetic properties (*χ*). The magnetic force field can be modified by changing the position or strength of the generating magnets, and the magnetic susceptibility can be modified by the choice of ferrofluid or its concentration or temperature (as the magnetic susceptibility of ferrofluids decreases significantly when heating).

### 2.2 Switching Between Stable and Unstable Regimes Using Localized Heating

**Figure 4:**
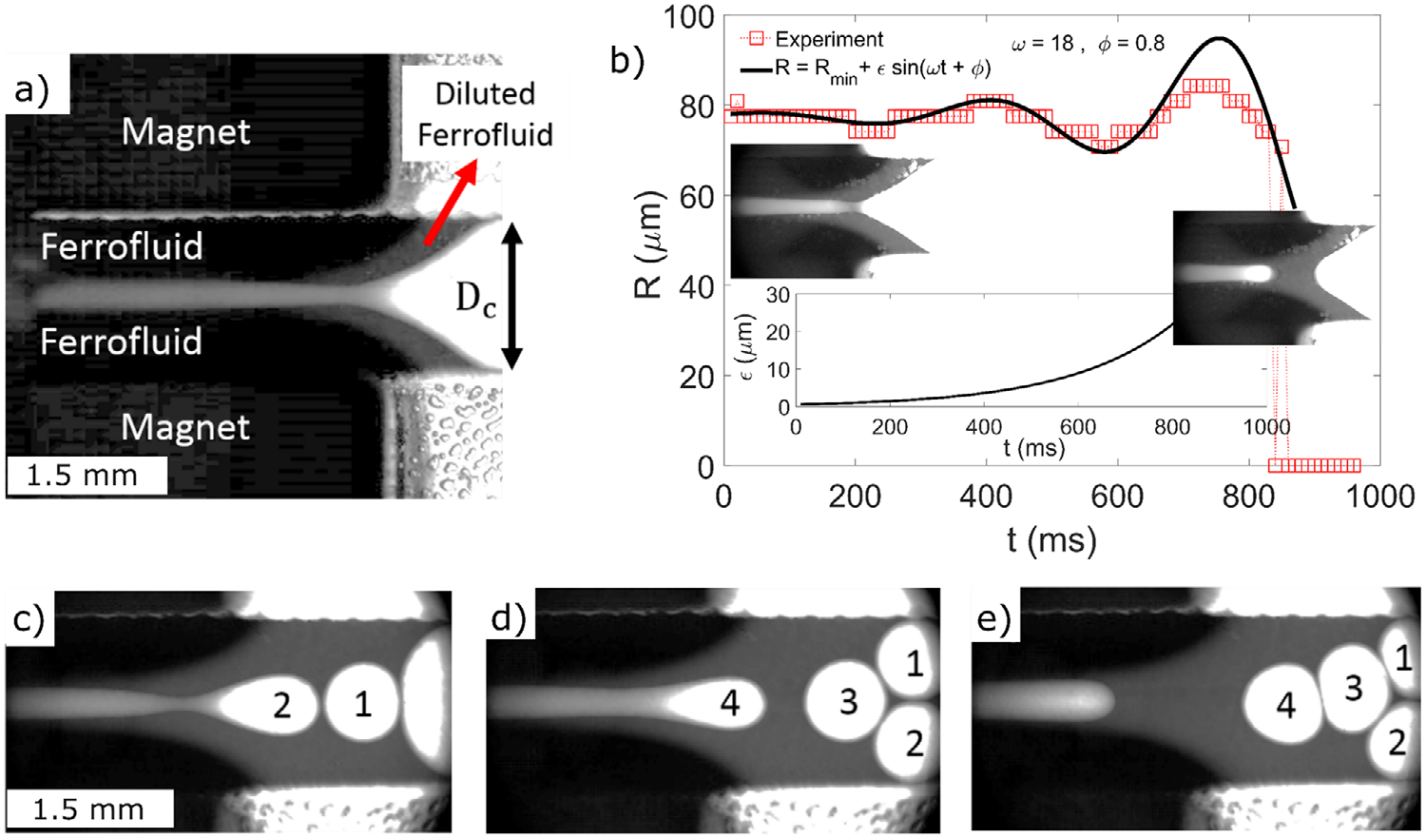
Toward droplet microfluidics. a) Flow of glycerol (bright), generating magnetically responsive Pickering‐type monodisperse droplets (flattened by dimension constraint). b) Increasing oscillatory instability and model fit (see main text for details), c–d) snapshots from Supporting Video V2, showing droplet formation. *D_c_* = 1.5 mm, glycerol flow rate is 2 µL/min and the ferrofluid is EMG905.

An external trigger of instability can be experimentally realized by optical means, using light irradiation to locally heat the ferrofluid. Better time‐resolved behavior can be obtained by means of optical microscopy. For this purpose, we designed a flow cell that deviates slightly from the cylindrical geometry by separating the generating permanent magnets more horizontally, keeping the ferrofluid lubrications to the lateral planes only, and allowing irradiation and detection along the vertical direction^[^ ^28^ ^]^ (the cavity is 3D printed with a width *D_c_* = 1.5 mm and height 280 µm with glycerol as a flowing liquid and EMG905 as ferrofluid). The evolution of the glycerol‐ferrofluid interface is then imaged under a Zeiss Axio zoom V16 microscope equipped with a Phantom v2511 camera.^[^ ^28^ ^]^ **Figure** **4a** shows the microfluidic flow behavior near the outlet (weakest point). When glycerol is pumped through this 2D magnetically confined flow channel, the magnetic forces stabilize the liquid‐in‐liquid flow (see Figure 4a) corresponding to *τ*(*k*) < 0. To destabilize the system (*τ*(*k*) > 0), we reduce the magnetic force (*χ*) by focusing the irradiation at *z* = *L* (see Video V2, Supplementary Video) to increase the local temperature and decrease the magnetic susceptibility.^[^ ^29^, ^30^ ^]^ This destabilizes the interface, which oscillates with exponentially increasing amplitude (see Figure 4b) with some ferrofluid drawn away (see Video V2, Supplementary Video). A fitting with the function *R* = *R_min_* + *ε*sin (*ω* *t* + *ϕ*) allows us to extract the exponential growth of disturbance (inset Figure 4b) as *ε* = *ε*_0_ *e* ^*τ* *t*^ = 0.6*e* ^4.5*t*^ with *τ* = 4.5/*s*. Comparing with Equation 2, the growth rate of the fastest growing wavelength *τ*(*k* = *k_max_*) with magnetic forces is $\tau_{m-max}=\frac{\sigma \beta_{min}{\left(1-Bo_{m}\right)}^{2}}{16\eta_{g}R_{min}}.$ At the onset of instability (*Bo_m_* ≤ 1) with *σ* ≈ 20 mN/m, *β*_*min*_ ≈ 200,^[^ ^23^ ^]^ *R_min_* = 74 µm *and* *η*_*g*_ = 1.1 Pa · s, we get 0.33/s < *τ*_*m* − *max*_ < 33/s *for* 0.99 < *Bo_m_* < 0.9. The dynamics hence, depend on the relative contribution of magnetic to surface energies and *τ* = 4.5/s is the right order of magnitude. Note that the difference in the growth rate is also due to geometric and boundary conditions effects which differ significantly for a planar lubricated geometry and an axisymmetric (cylindrical) lubricated case. The insets also show the system before and after the triggered instability. As illustrated in Video V2 (Supplementary Video), turning off the irradiation brings the system back to stability via oscillations of decreasing amplitude. To our knowledge, such a reversible feature (stable–unstable–stable) with an external stimulus is unique, within the approximation related to a slight loss of ferrofluid during the unstable process.

Tunable unstable flow can be mimicked by our setup, when taking advantage of the intrinsic ferrofluid properties, with a density of its component (magnetic nanoparticles) that increases with the magnitude of the external field.^[^ ^31^ ^]^ In our case, the end of the quadrupolar field generators exhibits a stray field of lower magnitude away from the magnets. This results in a lower magnetically loaded ferrofluid with a volume magnetic susceptibility lowered by dilution. Such a reduced magnetic force setup design is shown by a lower optical contrast in imaging the diverging end of the channel in Figure 4c–e. The reduced magnetic forces allow the generation of monodisperse droplets (Figure 4) of size 660 µm (flattened by the < 280 µm cavity height). Note that there is no surfactant in glycerol, and the stability results from the impenetrable ferrofluid layer formed between droplets (Figure 4c–e). Since ferrofluid is made of ferromagnetic particles of nanometric size, these drops could be treated as magnetic Pickering drops, where stability could be due to ferrofluid‐containing nanoparticles at the droplet surface. See Video V3 (Supplementary Video) for droplet generation, deformation and coalescence. The particularities of which must depend on the competition between Laplace pressure, wetting, particle adsorption energy and hydrodynamic drainage, which need further detailed attention^[^ ^32^, ^33^, ^34^ ^]^ and are a subject of further research.

## 3 Conclusion

Our work here delineates the stability limits of magnetically confined flows. We mitigate the failure mode of hydrodynamic flow focusing and explain the stability mechanism of liquid‐in‐liquid flow. The stability holds down to static conditions as there is no lower limit of flow rate. The stability condition of the magnetically confined liquid tubes is analogous to the stability of soft solid cylinders. Viscous forces play a key role in destabilizing this unique soft and slippery interface by deforming the interface.

We envisage that our strategy to build a stable fluid flow not bound by solid walls—with external and reversible control of its stability regime—opens new avenues for flow dynamics. While retaining the benefits (continuous jet, low shear) and overcoming the limitations (continuous sheath flow, chemical manipulation of transported liquid) of other approaches, the balance of magnetic and fluid forces forms a new pathway to stabilize complex “*soft and slippery*” structures, with applications of industrial, pharmaceutical and physiological relevance.

## 4 Experimental Section

### Magnetic Design and Encapsulation

The design principle of magnetically encapsulated flow channels involves balancing the magnetic pressure and Laplace pressure. It is reported in ref.[19] To create a ferrofluid‐encapsulated glycerol channel, a 3D‐printed cavity and permanent magnets fitted outside in a quadrupolar arrangement were used. Initially, the cavity was filled with glycerol and then ferrofluid was slowly introduced. The ferrofluid replaces glycerol at the wall of the 3D printed cavity due to the higher magnetic field. This creates a liquid‐in‐liquid tube. The volume of ferrofluid determines the size of the glycerol channel *R*_*0*_.

### X‐Ray Imaging

X‐ray absorption contrast imaging was performed. The pixel size of the detector is 20 µm. The sample with the ferrofluid encapsulated glycerol channel is placed over the detector and the system is exposed to X‐ray. The images were recorded and analyzed using ImageJ. The details of the X‐ray setup could be found in the article.^[^ ^19^, ^22^ ^]^

### Brightfield Imaging

Brightfield imaging was performed to capture the dynamics of the onset of instability. To do this, a 2D planar lubricated flow channel was designed. The details of the flow channel, the magnetic field design, imaging setup can be found in ref.[27] The setup was used for measuring velocity profiles in the glycerol channel in the stable limits and was later used to image the instability process.

### Material Properties

The interfacial tension between glycerol and various ferrofluids was measured, as shown in Figure 2. the Pendant drop method was used for interfacial tension. The magnetic susceptibility was measured using the induction method. The details of these measurements are provided in the article^[^ ^20^ ^]^ and a material property table is listed in the Section S4 (Supporting Information).

## Conflict of Interest

The authors declare no conflict of interest.

## Supporting information



Supporting Information

Supplementary Video V1

Supplementary Video V2

Supplementary Video V3

## Data Availability

The models and videos are freely available from https://github.com/hermanslab/SuppIntInstabil. Any other related data is available on request.
