# Supplementary material for: Suppressing Interfacial Instability of Immiscible Liquid‐in‐Liquid Flow Using Magnetic Forces
Source: Adv Sci (Weinh). 2025 Nov 7;13(4):e10327. doi: 10.1002/advs.202510327 (PMC12822428; doi:10.1002/advs.202510327)
Supplement: Supplementary file 1 — Supporting Information [file ADVS-13-e10327-s002.pdf]

Supplementary material: Suppressing interfacial instability of immiscible liquid-in-liquid flow using magnetic forces

Arvind Arun Dev[*1,2], Gholamhossein Bagheri[3], Eberhard Bodenschatz[3,4,5,6]
Thomas M. Hermans[*7] and Bernard Doudin[*1]

[1] Institut de Physique et Chimie des Matériaux de Strasbourg, UMR 7504 CNRS-UdS, 67034 Strasbourg, France
[2] Laboratoire Colloïdes et Materiaux Divises, CNRS UMR 8231, Chemistry Biology & Innovation, ESPCI Paris, PSL Research University, 10 rue Vauquelin, 75005 Paris, France.
[3] Laboratory for Fluid Physics, Pattern Formation and Biocomplexity, Max Planck Institute for Dynamics and Self-Organization, 37077 Göttingen, Germany,
[4] Institute for Dynamics of Complex Systems, University of Göttingen, 37077 Göttingen, Germany
[5] Laboratory of Atomic and Solid State Physics, Cornell University, Ithaca, NY 14853
[6] Sibley School of Mechanical and Aerospace Engineering, Cornell University, Ithaca, NY 14853
[7] IMDEA Nanociencia, C/ Faraday 9, 28049 Madrid, Spain.

arvind.dev@espci.psl.eu, thomas.hermans@imdea.org, bernard.doudin@ipcms.unistra.fr

Supporting Video captions

Supporting Video V1: Real-time footage of an oscillating flow experiment reaching up to 250 mL/min (water) flow rates. See Section S1 (below) for more detailed information.

Supporting Video V2: Real-time video of switching from a stable (i.e., uninterrupted jet) to unstable (i.e., droplet formation) flow upon light irradiation and subsequent heating. When turning the irradiation/heating off, the system returns to stability. See Supporting Section S3.1 for more information.

Supporting Video V3 Slowed down video (originally 150 frames per second) of droplet generation. See Supporting Section S3.2 for more details.

S1. Stability against oscillating flows

An oscillating pressure is imposed using an OB1 pressure-driven pump from Elveflow (Paris, France) with a time period of 10 s, 20 s and 40 s. Fig.S1 shows the pressure profile measured at the inlet of the flow channel. The antitube diameter is 2.4 mm and fluid is water. The ferrofluid used here is EMG905 (from FerroTec, USA). The cyclic images (Fig.S2) show the stages of oscillating flow due to oscillating pressure, shown by the water jet at the outlet (red line highlight the jet profile at outlet), see Video V1. No ferrofluid is sheared with these flow parameters, with peak flow rate during the cycle of $Q = 250$ mL/min.
EMG905 Ferrofluid is used here with large radius and high Reynolds numbers (> 2000). The flow is beyond the Stokes regime and the inertial effect becomes dominant, density becomes important and the stability limits require consideration of non-linear effects. Furthermore, at large R, the ferrofluid is saturated ($M = M_s$) with $M_s = 44$mT as the saturation magnetisation. This regime is outside the standard linear stability analysis and requires further attention.

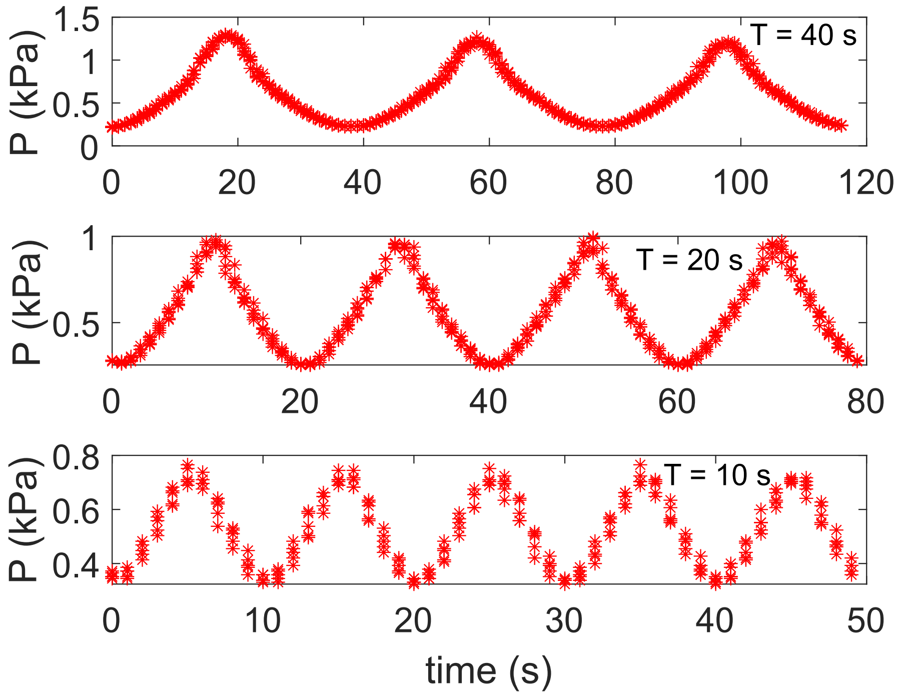

Figure S1: Pressure oscillations at the inlet of the antitube.

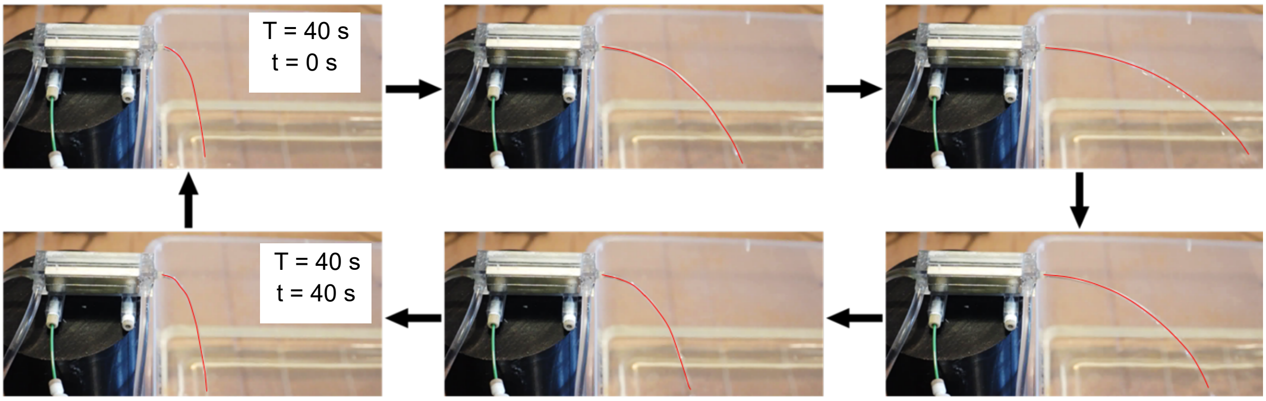

Figure S2: Oscillating flow out of antitube due to applied oscillating pressure at inlet.

S2. Instability

Consider a cylindrically lubricated liquid-in-liquid flo w of length L and radius of flo w R. The steady state axisymmetric flow is governed by the Navier-Stokes equation, in cylindrical coordinates for incompressible liquids with constant flo w rate. In the low Reynolds number limit where the viscous force dominates over the inertial force, this reduces to the Stokes equation, which reads

$$0 = -\frac{\partial P}{\partial z} + \eta\left(\frac{1}{r}\frac{\partial}{\partial r}(r\frac{\partial u}{\partial r})\right) \tag{1}$$

Integrating with respect to r we get for glycerol flow as

$$u_g = \frac{r^2}{4\eta_g}\frac{\partial P_g}{\partial z} + C_1 ln(r) + B \tag{2}$$

where u_g and η_g are the velocity of glycerol and viscosity, respectively. $\frac{\partial P_g}{\partial z}$ is the pressure gradient in the axial direction in the glycerol channel, C_1 and B are the constants of integration. At $r = 0$, $u_g(r)$ is finite so $C_1 = 0$

$$\frac{r^2}{4\eta_g}\frac{\partial P_g}{\partial z} + B = u_g(r) \tag{3}$$

For the ferrofluid using Eq. (1), we have

$$u_f = \frac{r^2}{4\eta_f}\frac{\partial P_f}{\partial z} + C ln(r) + D \tag{4}$$

To find the constants B, C, and D with the following boundary conditions

$$r = R + t_f, u_f = 0 \tag{5}$$

$$r = R, u_f = u_g \tag{6}$$

$$r = R, \eta_f\frac{\partial u_f}{\partial r} = \eta_g\frac{\partial u_g}{\partial r} \tag{7}$$

The ferrofluid volume is conserved and hence there is no net flow across any cross section.

$$Q_f = 2\pi \int_R^{R+t_f} r u_f dr = 0 \tag{8}$$

Using the boundary condition, we get

$$u_g = \frac{1}{4\eta_g}\frac{\partial P_g}{\partial z}\left[r^2 - R^2 + a_8\eta_r\right] \tag{9}$$

with $\eta_r = \eta_g/\eta_f$ is the viscosity ratio and the flow rate is given by

$$Q_g = -\frac{\pi}{8\eta_g}\frac{\partial P_g}{\partial z}R^4\beta \tag{10}$$

where $\beta = \left[1 - \frac{2\eta_r a_8}{R^2}\right]$ is the drag reduction factor with

$$a_8 = \frac{\left[R^2 + \frac{a_6 R^2}{4} - \frac{a_4 R^2}{4a_5} ln(R)\right]}{a_7} \tag{11}$$

$$a_7 = \left[1 - \frac{a_4}{8a_5}\right] \tag{12}$$

$$a_6 = \left[-\left(\frac{D_c}{R}\right)^2 + \frac{a_4}{a_5} ln\left(\frac{D_c}{2}\right)\right] \tag{13}$$

$$a_5 = \left[\frac{a_3}{2} - \frac{a_2}{2} ln\left(\frac{D_c}{2}\right)\right] \tag{14}$$

$$a_4 = \left[a_1 - \frac{a_2}{2}\left(\frac{D_c}{R}\right)^2\right] \tag{15}$$

$$a_3 = \left[\left(\frac{D_c}{2R}\right)^2 ln\left(\frac{D_c}{2}\right) - \frac{1}{2}\left(\frac{D_c}{2R}\right)^2 - ln(R) + \frac{1}{2}\right] \tag{16}$$

$$a_2 = \left[\left(\frac{D_c}{2R}\right)^2 - 1\right] \tag{17}$$

$$a_1 = \left[\left(\frac{D_c}{2R}\right)^4 - 1\right] \tag{18}$$

$$\frac{\partial P_f}{\partial z} = \frac{\frac{\partial P}{\partial z}}{a_7} \tag{19}$$

$$D = \frac{a_6}{16\eta_f}\frac{\partial P_f}{\partial z}R^2 \tag{20}$$

$$C = \frac{-a_4}{16a_5\eta_f}\frac{\partial P_f}{\partial z}R^2 \tag{21}$$

$$B = \frac{1}{4\eta}\frac{\partial P}{\partial z}\left[a8\eta_r - R^2\right] \tag{22}$$

The pressure gradient responsible for flow is given by [1]

$$\frac{\partial P_g}{\partial z} = \frac{\partial}{\partial z}\left(\int_0^{H_{max}} \mu_0 M dH\right) + \frac{\partial}{\partial z}\left(\frac{1}{2}\mu_0 M_I^2\right) + \frac{\partial}{\partial z}(\kappa\sigma) \tag{23}$$

Here P_g is the pressure in the glycerol which is also the excess pressure required to create the liquid-liquid interface [1]. $M(H)$ is magnetisation of the ferrofluid, σ is the interfacial tension between magnetic-nonmagnetic interface. M_I is the magnetisation at the magnetic-nonmagnetic interface. The first term is the fluid magnetic pressure and the second term is the magnetic normal traction at the magnetic-non-magnetic interface [2]. The last term is the Laplace pressure with κ as the curvature of the flow channel. The curvature of a deformed cylinder under small slope assumption

$$\kappa = \left(\frac{1}{R} - R''\right) \tag{24}$$

Here, $R' = \frac{\partial R}{\partial z}$ is the slope of the flow channel (glycerol channel) along the flow. The $\frac{\partial P_g}{\partial z}$ for the linear magnetic media is then given by using $M = \chi H$ in Eq. 23 gives

$$\frac{\partial P_g}{\partial z} = \frac{16\mu_0\chi(\chi+1)M_r^2 RR'}{\pi^2 w^2} - \sigma\left[\frac{R'}{R^2} + R'''\right] \tag{25}$$

Also we know from [3]

$$Q = \frac{-\pi\beta R^4}{8\eta_g}\frac{\partial P_g}{\partial z} \tag{26}$$

and for volume conservation, we have

$$\frac{\partial R}{\partial t} + \frac{1}{2\pi R}\frac{\partial Q}{\partial z} = 0 \tag{27}$$

Applying at the weakest point ($R = R_{min}$) and using the perturbation of kind $R = R_{min} + \epsilon(t)e^{ikz}$ with assumption $\frac{\epsilon}{R_{min}} << 1$, we note the evolution of $\epsilon(t)$. Where R_{min} is the minimum radius of glycerol flow (deformed weakest point) at the outlet (at $z = L$). Using Eq. 25, Eq. 27, Eq. 26 we get

$$\frac{\partial \epsilon}{\partial t} = \frac{\epsilon(t) R_{min} k^2 \beta_{min}\sigma}{16\eta_g}\left[1 - R_{min}^2 k^2 - \frac{16\mu_0\chi(\chi+1)M_r^2 R_{min}^3}{\sigma\pi^2 w^2}\right] \tag{28}$$

The solution is $\epsilon(t) \propto e^{t\tau(k)}$ where the growth rate is

$$\tau(k) = -\frac{R_{min}k^2\beta_{min}\sigma}{16\eta_g}\left[R_{min}^2 k^2 + \frac{16\mu_0\chi(\chi+1)M_r^2 R_{min}^3}{\sigma\pi^2 w^2} - 1\right] \tag{29}$$

If $\tau(k) < 0$, the system damps any perturbation, meaning it would lead to a stable interface. Since R_{min} and k are always positive, hence for stability $\tau(k) < 0$ for all the wave numbers ($k = 2\pi/\lambda$), if and only if

$$\frac{16\mu_0\chi(\chi+1)M_r^2R_{min}^3}{\sigma\pi^2w^2}-1>0 \tag{30}$$

Hence in the presence of a magnetic force, we have conditional stability if,

$$2R_{min}>\frac{1}{2^{\frac{1}{3}}}\left(\frac{\sigma\pi^2w^2}{\mu_0\chi(\chi+1)M_r^2}\right)^{\frac{1}{3}} \tag{31}$$

Note that Eq. 31 is similar to the stability condition derives for the Rayleigh Plateau instability of soft solid cylinder where stability requires $D>\frac{\sigma_s}{E}$. Here σ_s and E are the solid surface tension and elasticity respsctively [4, 5].

S2.1 Length and time scales

In the absence of magnetic term the system is unstable for small values of k, $\tau(k)>0$ (See Eq. 29), which is the classical Rayleigh-Plateau instability driven by finite interfacial tension. The length scale of the problem is then the fastest growing wavelength, given by the wavelength that maximizes $\tau(k)$ via $\tau_{max}=\tau_{k=k_{max}}$ with

$$\frac{\partial\tau(k)}{\partial k}=0. \tag{32}$$

Using Eq. 32 we get and Eq. 29

$$\lambda_{m-max}=\lambda_{max}/\sqrt{(1-Bo_m)}. \tag{33}$$

$$\lambda_{max}=2\pi R_{min}\sqrt{2}\approx 9R_{min} \tag{34}$$

Here λ_{m-max} and λ_{max} are the fastest growing wavelengths (length scales) with and without magnetic force, respectively. The timescale is given by $\Gamma_m=1/\tau(\lambda_{m-max})$ and $\Gamma=1/\tau(\lambda_{max})$ for with and without magnetic force respectively.

$$\Gamma_m=\frac{\Gamma}{(1-Bo_m)^2} \tag{35}$$

$$\Gamma=\frac{64\eta_gR_{min}}{\beta_{min}\sigma} \tag{36}$$

NOTE: It is evident from Figure 2 in the MS that the perturbation can be written as

$$R=R_{min}-cz+\epsilon(t)e^{ikz} \tag{37}$$

However, since $c=\frac{\partial R}{\partial z}$ is small, we treat the problem as local and neglect the linear slope. On the onset of instability, there is an intrinsic axial length scale given by λ_{m-max} and a scale for slope is $\approx k_{max}R_{min}$ with $k_{max}=\frac{2\pi}{\lambda_{m-max}}$. The ratio of the two slope scales can be written as $S=c/k_{max}R_{min}=c\sqrt{\frac{1-Bo_m}{2}}$. S can be a measure of the importance of c. Here, for the onset of instability (say $Bo_m\approx 0.9$), $S\approx c$ and c is experimentally of the order ≈ 0.02. Hence, we can neglect the linear term (cz), assuming small slope.

S3 Oscillating magnetic-nonmagnetic interface

S3.1 Unstable interface with growing distubances

The imaging was done at 100 fps, and a Canny edge detector was used to find the edges and

consequently find the evolution of the radius of flow (R_{min}) during the instability. We provide the raw as well as processed image for tracking of growing disturbances in R_{min}.

See Video V2 and note the following

1. Liquid-in-Liquid stable flow. Bright glycerol (center) and dark ferrofluid bands.

2. Upon illuminating the microfluidic channel with light from the source, we see the fluorescent particles in glycerol. The interface is stable.

3. On focusing light at the weakest point, the interface oscillates and the system destabilizes and some ferrofluid is taken away.

4. Switching off or de-magnifying the illumination, the interface again becomes stable.

This is possibly due to an increase in temperature when the light is focused in a small area and consequently, a decrease in magnetization of the magnetic fluids.

S3.2 Towards droplet microfluidics

See Video V3, and note the outlet is filled with some diluted ferrofluid (relatively brighter than the ferrofluid close to magnets).

1. The glycerol flow encounters a diverging flow channel near the end of the magnets, resulting in reduced/no magnetic forces (away from magnets). Hence necking takes place and droplets are formed. The glycerol flow rate is 2 μL/min and the ferrofluid is EMG905

2. When two droplets interact they do not coalesce, and a thin film of ferrofluid is present between them.

3. When the droplets push against each other, they deform significantly and coalesce.

S4 Material Property

Ferrofluid	Saturation magnetization M(mT)	Density 2 ρ(g/cc)	Interfacial tension σ (mN/m)	Magnetic Susceptibility χ
APG314	27.5	1.09	16.9 ± 0.84	0.515 ± 0.001
APGE32	33	1.15	28.4 ± 0.74	0.601 ± 0.001
APG1141	22	1.09	11.3 ± 0.56	0.364 ± 0.0001
EMG905	44	1.20	–	–

Table 1: Physical properties of ferrofluids used in the study.

References

[1] A. A. Dev, T. M. Hermans, and B. Doudin, "Ultra-soft liquid-ferrofluid interfaces," *Advanced Functional Materials*, vol. 34, no. 48, p. 2411811, 2024.

[2] R. E. Rosensweig, *Ferrohydrodynamics.* Courier Corporation, 2013.

[3] A. A. Dev, P. Dunne, T. M. Hermans, and B. Doudin, "Fluid drag reduction by magnetic confinement.," *Langmuir : the ACS journal of surfaces and colloids*, 2022.

[4] S. Mora, T. Phou, J.-M. Fromental, L. M. Pismen, and Y. Pomeau, "Capillarity driven instability of a soft solid," *Phys. Rev. Lett.*, vol. 105, p. 214301, Nov 2010.

[5] J. H. Snoeijer, "Analogies between elastic and capillary interfaces," *Phys. Rev. Fluids*, vol. 1, p. 060506, Oct 2016.
